# WIP Regulates Persistence of Cell Migration and Ruffle Formation in Both Mesenchymal and Amoeboid Modes of Motility

**DOI:** 10.1371/journal.pone.0070364

**Published:** 2013-08-07

**Authors:** Inmaculada Banon-Rodriguez, Julia Saez de Guinoa, Alejandra Bernardini, Chiara Ragazzini, Estefania Fernandez, Yolanda R. Carrasco, Gareth E. Jones, Francisco Wandosell, Ines Maria Anton

**Affiliations:** 1 Department of Molecular and Cell Biology, Centro Nacional de Biotecnología (CNB-CSIC), Madrid, Spain; 2 Department of Immunology and Oncology, Centro Nacional de Biotecnología (CNB-CSIC), Madrid, Spain; 3 The Randall Division of Cell and Molecular Biophysics, King's College London, London, United Kingdom; 4 Department of Molecular Neurobiology, Centro de Biología Molecular “Severo Ochoa” (CBM-UAM), Madrid, Spain; West Virginia University, United States of America

## Abstract

The spatial distribution of signals downstream from receptor tyrosine kinases (RTKs) or G-protein coupled receptors (GPCR) regulates fundamental cellular processes that control cell migration and growth. Both pathways rely significantly on actin cytoskeleton reorganization mediated by nucleation-promoting factors such as the WASP-(Wiskott-Aldrich Syndrome Protein) family. WIP (WASP Interacting Protein) is essential for the formation of a class of polarised actin microdomain, namely dorsal ruffles, downstream of the RTK for PDGF (platelet-derived growth factor) but the underlying mechanism is poorly understood. Using lentivirally-reconstituted WIP-deficient murine fibroblasts we define the requirement for WIP interaction with N-WASP (neural WASP) and Nck for efficient dorsal ruffle formation and of WIP-Nck binding for fibroblast chemotaxis towards PDGF-AA. The formation of both circular dorsal ruffles in PDGF-AA-stimulated primary fibroblasts and lamellipodia in CXCL13-treated B lymphocytes are also compromised by WIP-deficiency. We provide data to show that a WIP-Nck signalling complex interacts with RTK to promote polarised actin remodelling in fibroblasts and provide the first evidence for WIP involvement in the control of migratory persistence in both mesenchymal (fibroblast) and amoeboid (B lymphocytes) motility.

## Introduction

Dynamic remodeling of the actin cytoskeleton plays an essential role in cell motility [1]. Many actin-binding proteins that organise actin filaments into functionally specialized arrays such as filopodia, lamellipodia or ruffles are involved in cell displacement, contributing to individual amoeboid (rounded) or mesenchymal (elongated) migration [2]. It is often found that cytoskeletal proteins regulate the switch between both types of locomotion (e.g. GTPases [3]) or regulate one type but not the other (e.g. the actin filament crosslinker filamin is necessary for macrophage mesenchymal migration but dispensable for amoeboid migration [4]). Amoeboid locomotion is driven by the force generated via actin-mediated forward flow of the cell front, followed by actomyosin-mediated contraction of the mid region and rear uropod [5]. Mesenchymal movement is supported by strong integrin-mediated attachment at or just behind the leading edge and cell contractility that generates movement in a polarised morphology [6].

Platelet-derived growth factor (PDGF) is a chemotactic cytokine that induces rapid changes in cell shape associated with mesenchymal cell motility and migration [7]. PDGF exists as separate isoforms consisting of homo- or hetero-dimeric proteins of A- and B-polypeptide chains, which bind in a differential manner to two structurally related cell surface receptors, PDGFRα and PRGFRß [8]. The homodimer PDGF-AA binds exclusively the transmembrane tyrosine kinase receptor PDGFRα (PDGFRαα) whereas PDGF-BB (B chain homodimer) activates PDGFRαα, PDGFRαß and PDGFRßß [6]. Ligand binding induces dimerization of the receptors and subsequent transphosphorylation on specific tyrosine residues [7] that then become docking sites for proteins containing Src homology 2 (SH2) domains. These SH2-containing proteins either possess intrinsic enzymatic activity (e.g. phosphatidyl-inositol-3 kinase, PI3K) or act as adaptor proteins (e.g. Grb and Nck) that recruit other catalytically active signal transduction molecules to the receptor environment. Several distinct signalling cascades specific for the activated receptor tyrosine kinase (RTK), are then initiated that predominately converge on actin cytoskeleton remodelling pathways. The end point of these actin-linked cascades lead to the generation of filopodia, lamellipodia, peripheral membrane ruffles and circular dorsal ruffles. Circular dorsal ruffles, (also called waves, ring ruffles or actin ribbons), are highly dynamic surface structures that form transiently on the dorsal plasma membrane of adherent cells in 2D cultures and contribute to cytoplasmic remodelling, the establishment of polarity in motile cells, preparation of a stationary cell for subsequent movement, macropinocytosis and the internalization of cell surface receptors [9]–[11].

One of the pathways that regulate the formation of dorsal ruffles involves the Wiskott–Aldrich Syndrome protein (WASP) family proteins and the Arp2/3 (actin-related protein) complex that is activated by WASP proteins [12], [13]. The WASP family member N-WASP (neural WASP) has been localized to dorsal ruffles along with WIP (WASP Interacting Protein), dynamin 2, and cortactin after PDGF BB stimulation [14], [10]. N-WASP involvement in dorsal ruffle formation in mouse embryonic fibroblasts (MEFs) has been demonstrated through chemical inhibition with wiskostatin, siRNA treatment, or genetic depletion [15]. In addition, the expression of an N-WASP truncation mutant that cannot bind the Arp2/3 complex blocks the formation of these structures. The N-WASP/WIP complex is known to form a functional unit that contributes to actin cytoskeletal reorganisation and cell migration [16] but its contribution to ruffle formation has not been addressed. WIP is ubiquitously expressed and can independently bind filamentous actin (F-actin) [16], regulating at different levels the formation of most cellular actin-rich structures described to date including filopodia, lamellipodia, dorsal ruffles, stress fibres, podosomes and invadopodia [17], [18]. WIP overexpression in murine fibroblasts enhances dorsal ruffle formation in response to PDGF-BB stimulation [14]. Conversely, microinjection of anti-WIP antibody or the absence of WIP in murine null fibroblasts results in decreased ruffle formation in response to PDGF-BB treatment. Additionally, overexpression of a modified form of WIP lacking the actin-binding site blocks PDGF-BB-induced membrane ruffling [14]. WIP also interacts with other cytoskeletal-related proteins involved in dorsal ruffle formation such as cortactin, mAbp1 (murine actin-binding protein-1), and Nck [18].

Chemokines trigger amoeboid forms of cell movement in lymphocytes. This diverse family of small proteins all signal through G-protein coupled receptors (GPCR); the activated signalling cascades lead to actin cytoskeleton rearrangements and integrin activation to induce cell polarization and motility. WASP, exclusively expressed in immune cells, is involved in chemokine-mediated migration of distinct type of leukocytes. The lack of WASP impairs macrophage and dendritic cell adhesion and migration [19]–[21]. T cell and B cell migration in response to a chemokine gradient and lymphocyte trafficking in vivo are also affected by WASP deficiency [22]–[24]. The B cell immune response is delayed and reduced in the absence of WASP [25]; in addition, a role for WASP in B cell-mediated autoimmunity has been reported [26]. WIP is highly expressed in lymphoid tissue and protects WASP from degradation in resting cells [27], [28]. In fact, WIP null mice develop immune disorders that mimic Wiskott-Aldrich syndrome [29]. WIP deficient lymphocytes show defects in the subcortical actin filament network and in the normal T-cell responses to chemokines in vivo and in vitro [30], [22]. Nevertheless, the role of WIP in leukocyte amoeboid motility is poorly understood and the mechanisms underlying WIP function in cell movement remain elusive.

Here we demonstrate that WIP regulates the persistence of cell movement during both mesenchymal and amoeboid migration whilst cell speed is only affected in amoeboid B lymphocytes and not in fibroblasts using the mesenchymal mode of migration. Moreover we show that WIP binding to Nck is essential for fibroblast chemotaxis towards PDGF-AA.

## Materials and Methods

### Reagents and antibodies

Anti-GFP was purchased from Roche, anti-ßactin from Sigma, anti-GAPDH from AbD Serotec and anti-cortactin from Millipore. PDGFRα, WASP and N-WASP rabbit antibodies and monoclonal antibody to Nck were obtained from Santa Cruz Biotech. Rabbit anti-WIP was generated by ProteinTools [27]. Horseradish peroxidase (HRP)-labelled anti-mouse, anti-rat and anti-rabbit antibodies were purchased from Dako.

### Ethics statement

Animal experimentation was approved by the Ethical Committee of Animal Experimentation (CEEA-CNB) of Centro Nacional de Biotecnología (CNB-CSIC) and conforms to institutional, national and international regulations including the Royal Decree (RD 1201/2005).

### Derivation of murine primary fibroblasts

Lung pieces of wild-type or WIP^−/−^ SV129/BL6 mice were washed with PBS, minced and deposit into multi-6-well plates. Each small piece was squashed with a coverslip to favor tissue disaggregation and cell release, and cultured in fibroblast growth medium (DMEM medium supplemented with 10% FCS, penicillin and streptomycin (50 U/ml), 1X non essential aminoacids and 50 μM ß-mercaptoethanol) for 5 to 10 days. After removal of unattached debris, adherent cells were trypsinized and maintained in culture up to passage 12.

### B cells

Naïve B cells were freshly isolated from spleens of WIP^+/+^ and WIP^−/−^ mice, 2.5 months old, by negative immunoselection, as previously described (>95% purity; [31]). Purified B cells were labeled when indicated with 0.1 µm CFSE or SNARF-1 long-term dyes (Molecular Probes) for 10 min at 37°C before use. The murine 2PK3 B cell line was transiently transfected with the WIP-GFP construct, cloned in the pLV lentiviral vector, by electroporation. Cells were used for the experiments 24 h after electroporation.

### Transwell chemotaxis assay

Fibroblasts were incubated for 6–8 h with DMEM without serum. For transwell assays, 5×10^5^ starved cells in 500 μl of DMEM (Sigma-Aldrich) were added to the upper chamber of the transwell (Costar; 6,5 mm diameter, 8,0 μm pore size). The lower chamber was filled with 500 μl of DMEM supplemented with the indicated chemoattractant (15% FCS, 5 μM LPA, 50 ng/ml EGF, 20 ng/ml FGF, 1 ng/ml PDGF-BB or 50 ng/ml PDGF-AA). The cells were incubated overnight at 37°C. The upper chamber remaining cells were mechanically removed, and migrated cells through the membrane to the lower chamber were fixed with paraformaldehyde (PFA) 4%, and stained with DAPI. For the control transwells (without chemottractant added), the cells that were mechanically removed were the cells that migrate through the membrane. The chemotactic frequency, a measure of the specificity of migration, was calculated as follows: [(Number of cells migrating to the chemokines)/(Number of cells that stayed in the upper chamber in the control transwell)] ×100.

Freshly isolated or transduced B cells, 2.5×10^5^ cells in 100 μls of RPMI 10% FCS, were added to the upper insert of the Boyden chamber (Costar; 6,5 mm diameter, 3,0 μm pore size). The recombinant murine CXCL13 chemokine (Peprotech) was added at the indicated concentrations to the lower chamber filled with 600 μls of RPMI 10% FCS. After 2 h 30 min incubation at 37°C, we collected the volume of the lower chamber and counted the cells by flow cytometry. Migration frequency was estimated as the [(B cell n° at the lower chamber/the initial B cell input in the upper chamber) ×100] in each condition.

### Dunn chamber chemotaxis assays

Fibroblasts (6–8×10^4^ cells) were seeded on 18-mm square glass coverslips in fibroblast growth medium. Chemotaxis assays were carried out 12–24 h after seeding the cells; cells were starved of serum for 8 h prior to exposure to a serum gradient. Dunn chambers were set up as previously described [32] with DMEM 15% serum in the outer well. Cells were filmed at 37°C on Olympus IX50 Inverted microscopes fitted with phase-contrast optics, heated stages, and heated chambers. Frames were filmed using a CCD camera (Hitachi) every 5 min for 8 h using Acquisition Manager software from Kinetic Imaging (Wirral, UK) as previously described [32]. Cell tracks were generated from the time-lapse images using the image-processing program Lucida (kinetic Imaging), and the resulting tracks were analysed with the software Mathematica using long established in-house routines [33]. The mean migration speed and persistence for each tracked cell was calculated, and then the mean migration speed and persistence of the population derived. Cells that translocated less than 20 μm from their point of origin [34] were excluded from this analysis.

### B cell migration on planar lipid bilayers

The two-dimensional substrates based on the use of planar lipid bilayers containing GPI-linked ICAM-1 protein was prepared and assembled in FCS2 chambers (Bioptechs) as previously described [31]. Before imaging, the membranes were coated with 100 µM CXCL13 (Peprotech) for 30 min at RT. CFSE labeled WIP^+/+^ and SNARF-1 labeled WIP^−/−^ B cells in 1∶1 ratio were injected into the warmed (37°C) chamber; DIC, IRM and fluorescent images were acquired sequentially every 30 s for 20 min. All assays were done in PBS 0.5% FCS, 0.5 g/L D-glucose, 2 mM MgCl_2_, and 0.5 mM CaCl_2_. Images were acquired on a Zeiss Axiovert LSM 510-META inverted microscope with a 40× oil-immersion objective, and analysed with Imaris 6.0 software (Bitplane). Graphs and statistical analysis were done with Prism 4.0 software (GraphPad).

### Western blot analysis

Growing or infected fibroblasts plated in p100 dishes were washed in PBS, lysed in cold lysis buffer containing 0.2% Triton X-100, 150 mM NaCl, 50 mM Tris-HCl pH 7.4, 1 mM EDTA, 1 mM EGTA, with protease (Complete from Merck) and phosphatase inhibitors (50 mM NaF, 1 mM Sodium orthovanadate and 1 mM okadaic acid), and scraped off. Lysates were centrifuged at 13,000 rpm to spin down cell debris. Similarly, freshly isolated and transduced B cells were lysed in cold lysis buffer for 30 min and spun at 13,000 rpm for 30 min; the fractions of soluble proteins were collected. Soluble proteins were analysed by SDS-PAGE and Western blot. In brief, proteins were separated by gel electrophoresis under denaturing and reducing conditions, then, separated proteins were electrophoretically transferred to nitrocellulose or PVDF membranes using a Bio-Rad Mini protein II transfer apparatus. Blots were blocked with 5% dried milk solution diluted in TBS-T (10 mM Tris-HCl, pH 7.5, 100 mM NaCl, 0.1% Tween 20) containing NaF 5 μM for 1 h at room temperature (RT), and incubated overnight at 4°C with the primary antibody diluted in the same buffer. Labeling was detected by incubation with HRP-conjugated secondary antibodies (diluted in TBS-T) for 1 h at RT and enhanced chemiluminiscence (ECL) detection system.

### Infection using lentiviral vectors

Recombinant lentiviral stocks were produced in 293T cells by co-transfecting the transfer vector (GFP, WIP-eGFP, WIPΔNBD or WIPΔWBD,) the envelope plasmid pMD.2G, and the packaging plasmid pCMVR8.91, as previously described [35]. Cells (1.5×10^7^) were seeded onto 150 cm^2^ flasks and transfected with 10 μg DNA envelope, 30 μg DNA packaging and 40 μg DNA transfer vector by precomplexing with 0.125 mM PEI (22 kDa) for 15 min at RT in OptiMEM. After 4 h at 37°C the medium was replaced with fresh DMEM 10% FCS and virus particles were harvested 48 and 72 h post transfection. After filtering through a 0.45 μm-pore-size filter, the virus suspension was concentrated by centrifugation at 50,000 g for 2 h at 4°C. The resulting pellet was resuspended in RPMI (Sigma, UK) and stored at –80°C until used. The desired number of fibroblasts were plated in complete culture medium and concentrated lentivirus was added to the cells at a multiplicity of infection (MOI) of 10 and incubated for 72 h to allow maximal expression of recombinant proteins before being used for experiments. For B cell transduction, 2×10^6^ purified B cells (none-labelled or SNARF-1 labelled) were infected with concentrated lentivirus (MOI of 1–10) in 500 μls of RPMI with 10% FCS and LPS (2.5 μg/ml; Sigma) for 6 h at 37°C; then, the medium was replaced and the infected B cells were cultured for 24 or 48 h to allow protein expression.

### Immunofluorescence

The cells were fixed in 4% PFA in PBS, permeabilised with 0.5% Triton X-100 in PBS, blocked with 3% bovine serum albumin (BSA) in PBS+0.1% Tween-20 and incubated with appropriate primary and secondary antibodies or fluorescent phalloidin diluted in PBS+0.1% Tween-20. Coverslips were mounted onto slides using Vectashield mounting medium (Vector Laboratories, UK) and visualised using a Zeiss LSM 510 Meta confocal laser scanning head attached to a Zeiss Axioplan 2 microscope. LSM 510 software was used to obtain merged confocal images.

In the case of B cells, they were fixed in 4% PFA (10 min, 37°C) after 30 min in contact with the planar membranes, permeabilized with 0.2% Triton X-100 in PBS (5 min, RT), blocked with PBS with 2% BSA and 2% FCS, stained with Alexa-Fluor647-Phalloidin (20 min), and imaged on a Zeiss Axiovert LSM 510-META inverted microscope with a 40× oil-immersion objective. Data analysis was done with Imaris 6.0 software (Bitplane).

### Flow cytometry

The cells were either grown on complete medium or starved for 24 h and stimulated with 50 ng/ml of PDGF-AA for different periods of time. After stimulation, cells were washed once with PBS, trypsinised and resuspended at 10^6^ cells/100 μl. Cells were centrifuged, fixed with 4% PFA for 20 min and resuspended in PBS staining (PBS with 3% FCS and EDTA 2mM). The antibody specific for the extracellular region of PDGFRα was diluted in PBS staining, and cells were incubated for 1 h with the primary antibody and for 30 min with the secondary. Finally, cells were resuspended again in PBS staining and analysed by flow cytometry in a FACScalibur cytometer (Becton Dickinson).

Freshly isolated B cells (2×10^5^) were stained with fluorescently conjugated rat anti-mouse CD19 and rat anti-mouse IgM (Pharmingen) for 30 min on ice, washed, and analysed by flow cytometry as above.

### Statistical analysis

The unpaired Student *t* test was applied to calculate statistical significance, except for the determination of the confidence interval of mean direction of migration (where the Rayleigh's test was applied) and for comparison of ruffle formation in transduced populations (where the ANOVA test and test of Tukey were applied).

## Results

### WIP deficiency impairs amoeboid cell motility

To investigate the role of WIP in amoeboid migration, we studied B lymphocyte behaviour in response to the most specific B cell chemokine CXCL13 (C-X-C motif chemokine 13). We isolated B cells from spleens of control (WIP^+/+^) and WIP^−/−^ mice. B cell phenotypic analysis showed no significant differences in cell size, cell complexity, or surface expression of typical markers for these lymphocytes (CD19, IgM) (Figure S1a). We detected low WASP level in WIP^−/−^ B cells, as predicted from the reported role of WIP in protecting WASP from degradation [27], [28] (Figure S1b). More surprisingly, low Nck levels were also recorded in WIP^−/−^ B cells (Figure S1b).

**Figure 1:**
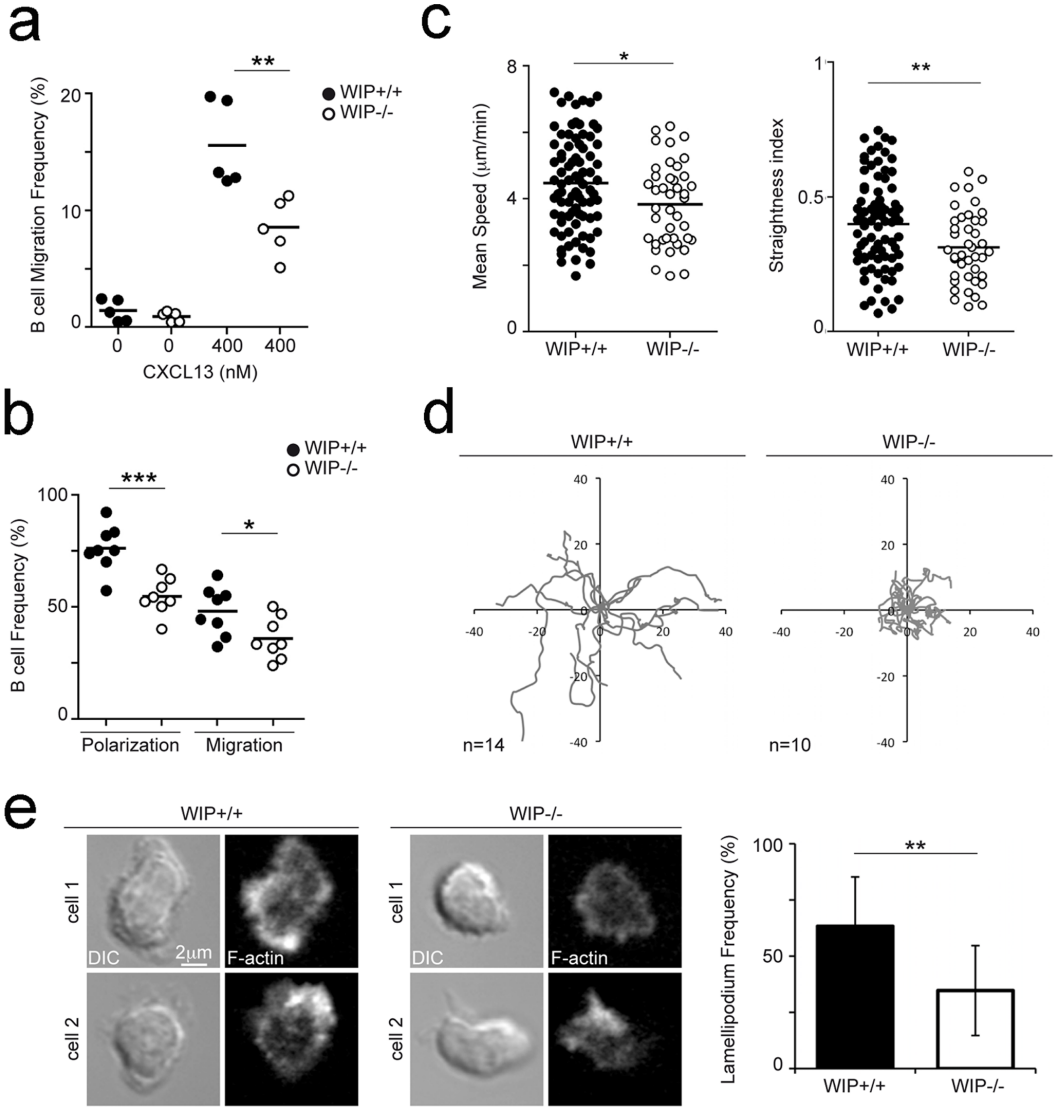
The lack of WIP reduces B cell amoeboid motility. **a** Chemotactic response of freshly isolated WIP^+/+^ and WIP^−/−^ B cells to CXCL13 (400 nM) in Boyden chambers. **b** Polarization and migration frequencies of WIP^+/+^ and WIP^−/−^ B cells settled on ICAM-1-containing planar membranes coated with CXCL13. In (**a**) and (**b**) each dot corresponds to one experiment; black thick bar, averaged value. **c** Mean speed values (left panel) and Straightness index (right panel) of WIP^+/+^ and WIP^−/−^ B cells in the same conditions than in (**b**); each dot corresponds to a single cell. **d** Tracks of representative WIP^+/+^ and WIP^−/−^ B cells migrating on the planar membranes; each line corresponds to a single cell track. **e** DIC and F-actin images of representative WIP^+/+^ (left panels) and WIP^−/−^ (middle panel) B cells; right panel, lamellipodium frequency in control and deficient B cells. Data on (**c**) and (**e**) is the merge of three experiments. *, p<0.05; **, p<0.001; ***, p<0.0001.

We evaluated the B cell chemotactic response to a gradient of CXCL13 in Boyden chambers; the absence of WIP reduced by half the percentage of migratory B cells, independently of the chemokine dose used (400 nM Figure 1a; 40 nM data not shown). To track B cell motility in real time, we used a 2D system based on the use of artificial planar lipid bilayers containing the adhesion molecule ICAM-1 (intercellular adhesion molecule-1) and CXCL13 coating [31]. The presence of adhesion molecules and a homogeneous chemokine coating results in a haptokinesis system, where B cells move by random migration. We labelled control and WIP^−/−^ purified B cells with distinct fluorescent probes (CFSE, SNARF-1), and seeded cell aliquots onto the haptokinetic substratum. CXCL13 signalling promoted B cell polarization, as measured by the formation of dynamic membrane protrusions from the cell body over time (detected by Differential Interference Contrast, DIC, microscopy) (Video S1). The chemokine-activated LFA-1/ICAM-1 interaction that supports cell migration was followed by Internal Reflection Microscopy, IRM) (Video S1). WIP^−/−^ B cells showed a significant reduction in polarization and migration compared with wild-type B cells (Figure 1b). They displayed lower mean velocity and directionality (measured as a persistence index using Imaris software, Bitplane) than WIP^+/+^ counterparts (Figure 1c). In addition, WIP^−/−^ B cells displayed more restricted tracks than control B cells (Figure 1d).

The defects in B cell migration due to the lack of WIP may be related to alterations in actin polymerization. We evaluated F-actin rich lamellipodia by phalloidin staining in fixed B cells attached to the 2D substratum. WIP^−/−^ B cells had fewer F-actin rich lamellipodia than WIP^+/+^ counterparts (Figure 1e). We tracked WIP localization at the B cell-substratum contact plane in real time by expression of a WIP-GFP fusion protein in the B cell line 2PK3. We observed that WIP accumulated at the lamella of new forming membrane ruffles, where nascent adhesion points also appeared (detected by IRM) (Figure S2 and Video S2).

**Figure 2:**
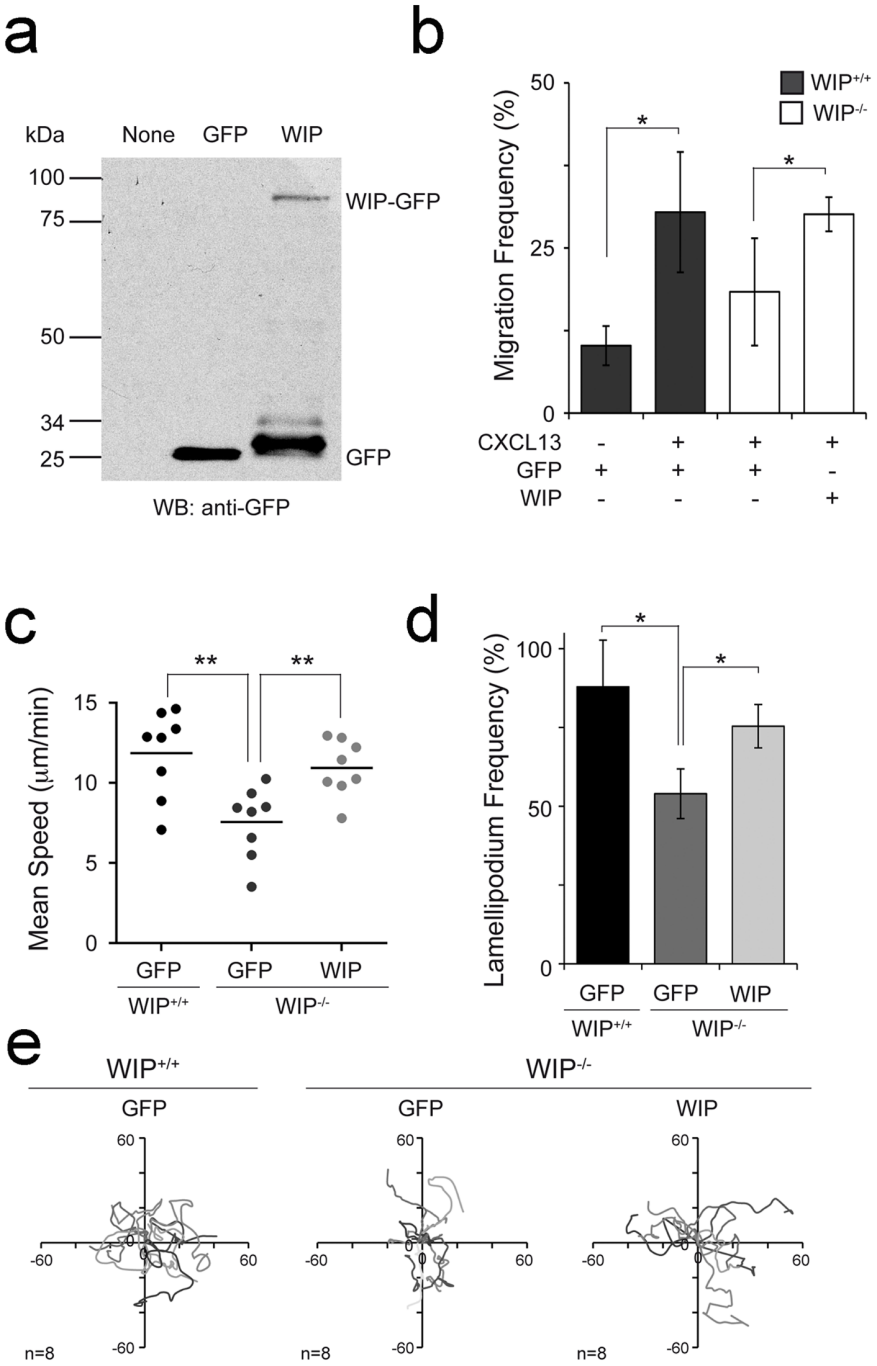
WIP re-expression recovers amoeboid motility in WIP ^−**/**−^ **B cells.** Purified WIP^+/+^ and WIP^−/−^ B cells were transduced with recombinant lentivirus expressing GFP (GFP) or full-length WIP-GFP (WIP) as described in Materials and Methods; 24 h later, they were used for the different assays. **a** Total lysates of the indicated transduced B cells were used to detect the expression of the WIP construct by western-blot with anti-GFP. **b** Migration frequency of the indicated transduced B cells in response to 400 nM CXCL13 in Boyden chambers; data merged from two experiments are shown. **c** Mean speed values, **d** F-actin-rich lamellipodium frequency, and **e** representative tracks of the specified transduced B cells migrating on the planar membranes; each dot is a single cell in (**c**); data from a representative experiment are shown in (**e**). *, p<0.05; **, p<0.001.

To confirm the relevance of WIP function in amoeboid cell motility, we reconstituted purified WIP^−/−^ B cells with full length WIP as GFP fusion protein by transduction with recombinant lentivirus; we used GFP-expressing recombinant lentivirus as a transduction control (Figure 2a). We observed that lentiviral infected B cells displayed enhanced basal migration and also CXCL13-mediated chemotaxis in Boyden chambers when compared with freshly isolated B cells (Figures 1a, c and 2b, c). The reconstitution of WIP^−/−^ B cells with full length WIP recovered chemotaxis, mean velocity values and F-actin-rich lamellipodium frequency to the levels of GFP-transduced control B cells (Figure 2b–d); the tracks were similar to those seen in GFP-transduced control B cells (Figure 2e). In contrast, WIP^−/−^ B cells transduction with GFP lentivirus did not rescue chemotaxis, mean speed or lamellipodium production frequency; these B cells still showed restricted tracks (Figure 2b–e).

These findings identify a role for WIP in B lymphocyte motility and stress the importance of WIP function in F-actin-rich lamellipodium formation and amoeboid motility where it regulates polarization, speed and directional persistence of migration.

### WIP-null fibroblasts retain a normal phenotype

**Figure 3:**
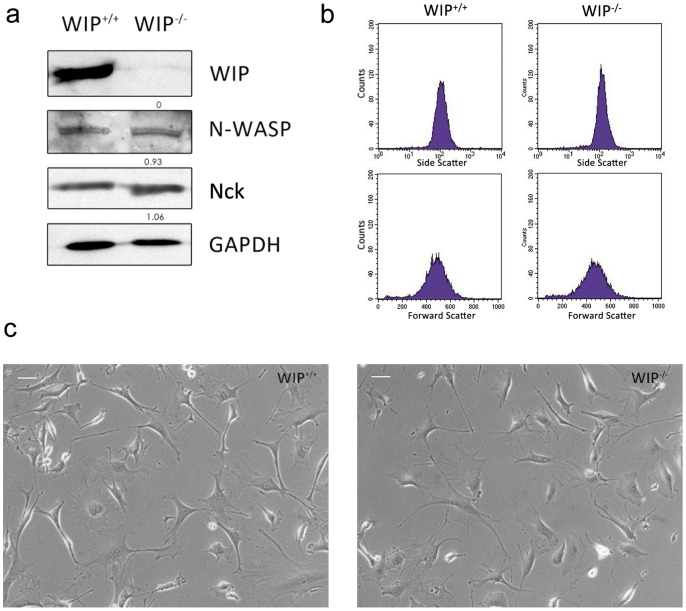
WIP deficiency does not affect fibroblast morphology or protein content. **a** Representative western blot of WIP, N-WASP and Nck expression in soluble lysates of lung-derived fibroblasts from WIP^+/+^ and WIP^−/−^ mice. Numbers indicate relative expression levels of each protein to GAPDH content and control fibroblasts determined by densitometry. GAPDH labeling confirmed equivalent protein loading control. **b** FACS analysis of forward and side scatter in control (WIP^+/+^) and WIP^−/−^ fibroblasts. **c** Phase-contrast images from plated control (WIP^+/+^) and WIP^−/−^ murine fibroblasts do not show evident morphological differences between both populations. Scale bar 50 µm.

In order to address the role of WIP in mesenchymal cell movement, we examined fibroblast migration in response to growth factors. Primary lung fibroblasts were derived from control (WIP^+/+^) or WIP^−/−^ adult mice and maintained in complete medium with fetal calf serum (FCS) up to a maximum of 12 passages to prevent undesired cell selection. Absence of WIP was confirmed by Western blot analysis of soluble cell lysates (Figure 3a). Neither the levels of WIP- associated proteins such as N-WASP or Nck, nor those of unrelated cytoskeleton-associated proteins such as GAPDH, were affected by the absence of WIP. Similarly, no significant differences were observed between cell size or cell surface rugosity of control and WIP^−/−^ cells when both populations were analysed by flow cytometry (Figure 3b). In order to confirm that WIP deficiency did not alter cell morphology, we performed image comparison of growing fibroblasts in 2D culture. Control and WIP^−/−^ fibroblasts showed comparable morphologies in vitro (Figure 3c). These results indicate that WIP deficiency is not sufficient to modify fibroblast morphology or the levels of N-WASP and Nck.

### WIP controls fibroblast chemotaxis through controlling directional persistence

**Figure 4:**
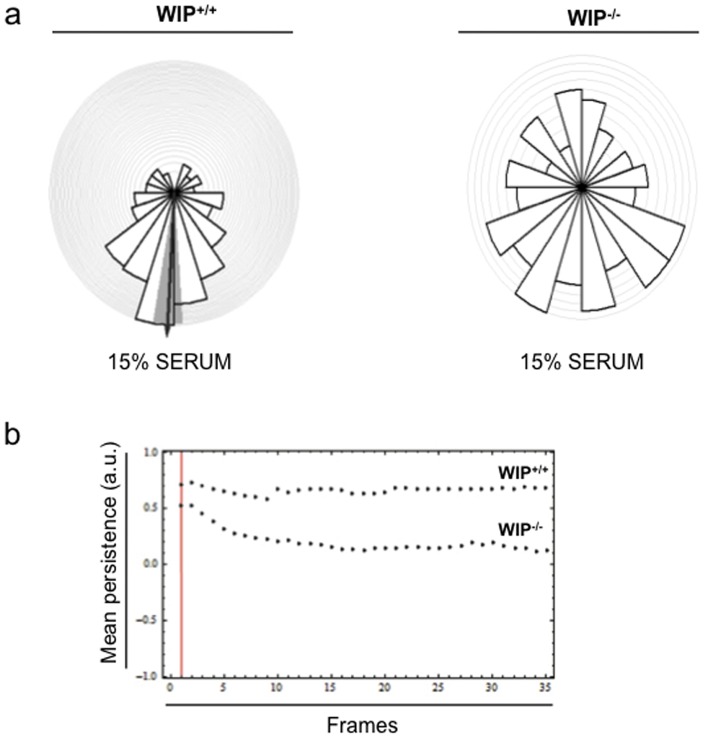
Chemotacting WIP ^−**/**−^ **fibroblasts towards serum show reduced persistence.** Control (WIP^+/+^) and WIP^−/−^ murine fibroblasts were assayed for chemotaxis towards 15% serum in Dunn chambers. **a** Circular rose plots show the proportion of cells with migratory direction lying within each 20° interval (serum source at bottom of histogram). The arrow represents the mean direction of migration; the grey segment represents the 95% confidence interval determined by the Rayleigh's test (p<5,67×10^−12^) between WIP^+/+^ and WIP^−/−^ murine fibroblasts. **b** Representation of mean persistence values. Vertical red line represents the onset of image acquisition, once the gradient has been formed. Arbitrary units (a.u.).

WIP is known to be required for lymphocyte chemotaxis to CXCL12 both *in vitro* and *in vivo* [22]. More recently, WIP's contribution to fibroblast chemotaxis has been reported [36]. However, the mechanism by which WIP modulates cellular responses to chemotactic stimuli remains largely unsolved. To address this question, we loaded Dunn chemotaxis chambers with 15% FCS in the external ring and imaged by phase contrast time-lapse microscopy the displacement of fibroblasts derived from control or WIP^−/−^ murine lungs [37]. An in-house (Dunn & Jones) Mathematica 6.0 (Wolfram Research Institute) workbook analysis of cell tracks over time demonstrated that WIP^+/+^ fibroblasts (n = 167) migrated preferentially towards the chemoattractant source while WIP^−/−^ fibroblasts (n = 154) showed random motility (Figure 4a, Figure S3 and animations WIP^+/+^ (Video S3) and WIP^−/−^ (Video S4)). Interestingly, both cell types had similar mean velocity: 0.44±0.01 μm/min for control fibroblasts and 0.41±0.01 μm/min for WIP^−/−^ fibroblasts (Figure S3b). In contrast, WIP deficiency significantly decreased cell persistence during chemotaxis (Figure 4b and Figure S3c). These results indicate that WIP does not regulate fibroblast displacement velocity but it is necessary to maintain directional persistence during fibroblast movement towards a source of serum-derived chemoattractants, as also observed for amoeboid cell migration.

**Figure 5:**
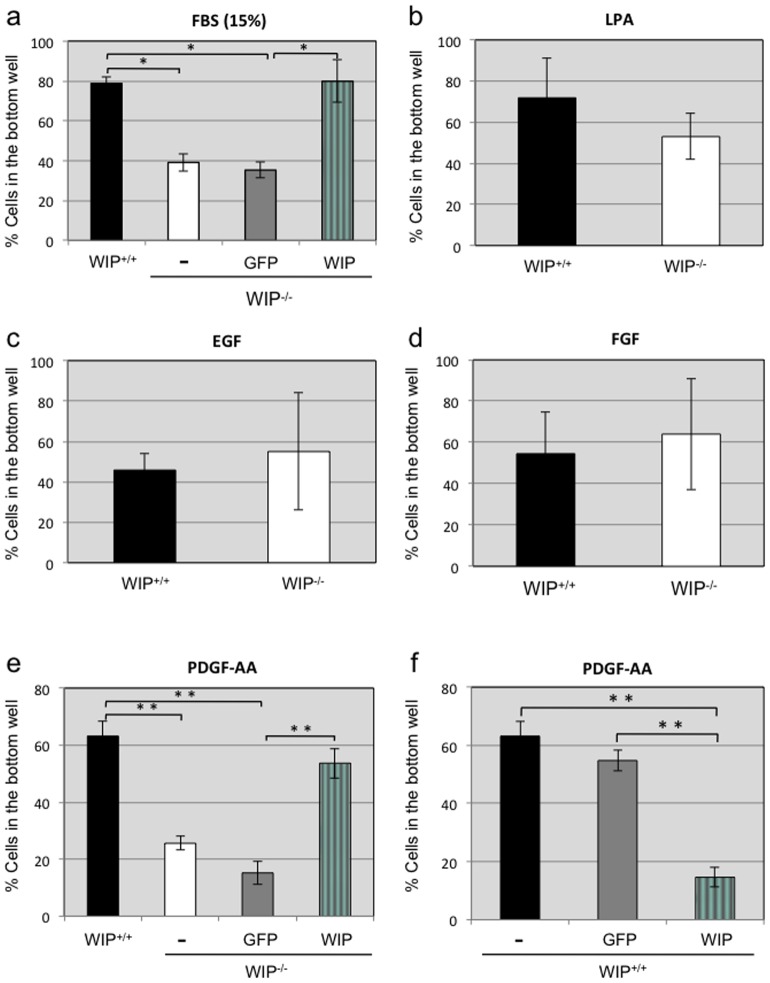
Reduced chemotaxis towards serum or PDGF-AA of WIP ^−**/**−^ **murine fibroblasts.** Control (WIP^+/+^) and WIP^−/−^ murine fibroblasts were loaded onto the upper chamber of the Transwell and the percentage of cells chemotacting to the lower well was calculated. Cells were assayed for chemotaxis towards 15% serum (**a**), 5 µM LPA (**b**), 50 g/ml EGF (**c**), 20 ng/ml FGF (**d**) and 50 ng/ml PDGF-AA (**e** and **f**). Reconstitution assays were performed by lentiviral-mediated expression of WIP-GFP or control GFP in WIP^−/−^ cells and overexpression assays by transduction of control (WIP^+/+^) fibroblasts with GFP or WIP-GFP. * p<0,05; ** p<0,001.

To confirm the function of WIP in fibroblast chemotaxis towards serum, we performed Boyden chamber (Transwell) analyses with control and WIP^−/−^ fibroblasts. Following the pattern seen in the Dunn chamber, migration towards the serum loaded into the lower chamber was significantly decreased in WIP^−/−^ fibroblasts compared to control cells (Figure 5a). Significantly, chemotaxis was restored in WIP^−/−^ fibroblasts rescued by exogenous WIP-GFP expression but not by GFP alone (Figure 5a). This result supports the conclusion that the diminished chemotaxis observed in WIP^−/−^ fibroblasts was due to the absence of WIP and not to potential secondary effects derived from genetic manipulation.

### WIP is necessary for fibroblast chemotaxis towards PDGF-AA

Serum contains an array of lipid and peptide chemoattractants, with LPA (lysophosphatidic acid), EGF (epidermal growth factor), FGF, (fibroblast growth factor) and PDGF (platelet-derived growth factor) being some of the well-known constituents [34]. To determine whether the contribution of WIP to the observed chemotaxis was exclusively induced as a response to a combination of chemotactic cues or was specific to some of them, we independently tested the responses of WIP^−/−^ fibroblasts to each of the above mention chemoattractants (Figure 5b–e). WIP-deficiency did not modify fibroblast chemotaxis towards LPA (5 μM; 2 μM, data not shown), EGF (50 ng/ml), FGF (20 ng/ml) (Figure 5b–d) or a combination of the two latter proteins (data not shown). In contrast, WIP^−/−^ fibroblasts showed a significantly reduced capacity to migrate towards PDGF-AA (Figure 5e, 50 ng/ml; 10 ng/ml, data not shown). This deficiency was recovered by exogenous expression of WIP-GFP but not of control GFP (Figure 5e). We then posed the question of whether WIP overexpression would increase the chemotactic response. Control murine fibroblasts were transduced with lentivirus directing GFP (control) or WIP-GFP expression (4- to 6-fold endogenous WIP expression; data not shown) and their migration towards PDGF-AA was quantified (Figure 5f). WIP overexpression led to a significantly reduced capability of the murine lung fibroblasts to reach the bottom well of the Transwell. These results indicate that WIP is necessary for fibroblast chemotaxis induced by serum and PDGF-AA, and that fine-tuned WIP levels are required for proper control of the process since both reduced and increased WIP expression impair chemotaxis.

### WIP expression is required for dorsal ruffle formation

**Figure 6:**
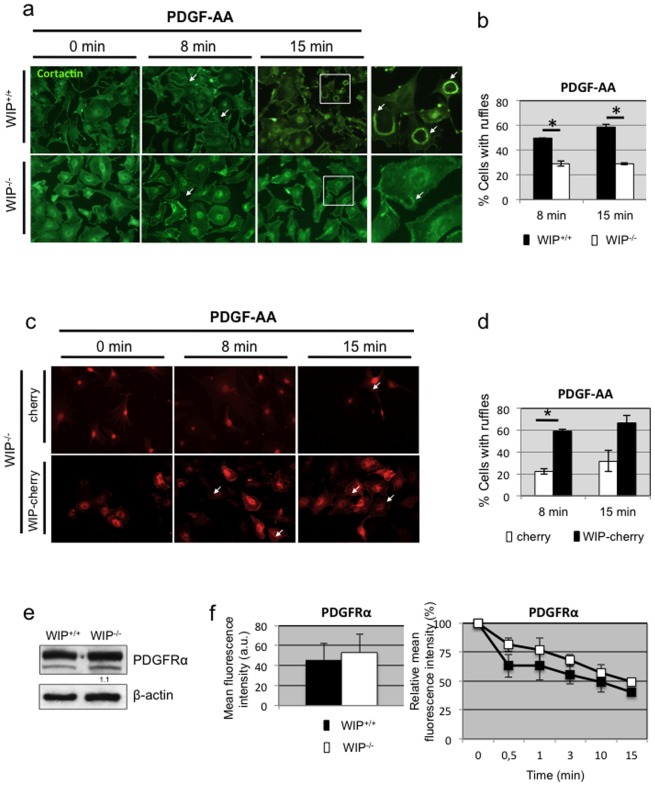
PDGF-AA-induced dorsal ruffle formation is diminished in WIP ^−/−^ fibroblasts. **a** Control (WIP^+/+^) and WIP^−/−^ primary murine fibroblasts were serum starved over night (0 min) or serum starved and stimulated with PDGF-AA for increasing times (8 and 15 min). Fixed and permeabilised cells were stained with anti-cortactin and FITC-secondary antibody and imaged in a Zeiss microscope to identify dorsal ruffles (white arrow). Magnifications of the boxed areas are shown as right panels. **b** The percentage of WIP^+/+^(black) and WIP^−/−^ (white) cells forming dorsal ruffles after PDGF-AA stimulation is plotted against incubation times. **c** WIP^−/−^ primary fibroblasts were lentivirally transduced to express control cherry or WIP-cherry, starved and incubated with PDGF-AA for 8 or 15 min. Fixed cells were imaged. **d** The percentage of WIP^−/−^ cells expressing cherry (white) or WIP-cherry (black) and forming dorsal ruffles after PDGF-AA stimulation is plotted against incubation times. **e** Representative western blot of PDGFRα expression in soluble lysates of lung-derived fibroblasts from WIP^+/+^ and WIP^−/−^ mice. Numbers indicate relative expression levels of the protein to control fibroblasts determined by densitometry. ß-actin labeling confirmed equivalent protein loading control. **f** WIP^+/+^ (black) and WIP^−/−^ (white) primary fibroblasts were grown in the presence of serum (left panel) and stained with anti-PDGFRα plus labeled secondary antibody and analysed by FACS. The mean fluorescence intensity of positive cells in both populations is represented. Right panel, WIP^+/+^ (black) and WIP^−/−^ (white) primary fibroblasts were starved (time 0) and stimulated with PDGF-AA and stained as above. Percent of mean fluorescence intensity relative to starvation is represented. Arbitrary units (a.u.). * p<0,05.

Having identified a role for WIP in PDGF-AA chemotaxis in fibroblasts we subsequently studied whether WIP contributes to dorsal ruffle formation after PDGF-AA stimulation. Dorsal ruffles were identified as dynamic and transient circular structures enriched in cortactin (Figure 6a) and polymerized actin (Figure S4a). Serum-starved WIP-null fibroblasts showed impaired dorsal ruffle formation in the presence of PDGF-AA at all times tested, the percent of WIP^−/−^ cells with ruffles is 2 fold less than in control cells after 15 min exposure to the cytokine (Figure 6b). The decreased ability of WIP-deficient fibroblasts to form dorsal ruffles after PDGF-AA exposure was restored after lentiviral expression of WIP-cherry in WIP^−/−^ fibroblasts but not after lentiviral expression of a control cherry construct (Figure 6c, d and S4b). Moreover, WIP-cherry redistribution to dorsal ruffles after PDGF-AA treatment (Figure 6c) emphasizes the crucial role of WIP in dorsal ruffle dynamics.

### The receptor for PDGF-AA is not affected by the absence of WIP

Of the different homo- or heterodimeric PDGFRs expressed on the cell surface, PDGF-AA exclusively activates the homodimeric PDGFRαα isoform as shown *in vitro* and *in vivo* [38]. One possible explanation of the limited responses we observed in WIP^−/−^ cells would be a reduction in PDGFRα levels in WIP^−/−^ fibroblasts. To test this hypothesis we performed Western blot analysis of total protein extracts obtained from WIP^+/+^ and WIP^−/−^ fibroblasts (Figure 6e) and performed real time PCR and microarray analyses from RNA samples (data not shown). We found no differences in the levels of PDGFRα in any of the assays. As measured by FACS, there was also no significant difference in the cell surface levels of PDGFRα between WIP^+/+^ and WIP ^−/−^ cells, neither in the presence of serum (Figure 6f, left panel) nor after serum starvation (Figure 6f, right panel).

PDGFRα is known to undergo endocytosis upon treatment with PDGF-AA [38] so we speculated that WIP could instead have a role in regulating PDGF-mediated signalling at this level. Analyses of surface levels of PDGFRα over a time course of treatment with PDGF-AA revealed that WIP^−/−^ cells showed no difference to control ones in their endocytic capacity for PDGFRα (Figure 6f right panel). These results rule out the possibility that WIP could modify PDGFRα expression, surface distribution or endocytosis rate at the times tested. These data suggest an alternative downstream role for WIP as a scaffolding/signalling molecule.

### WIP binding to Nck and N-WASP is required for dorsal ruffle formation

**Figure 7:**
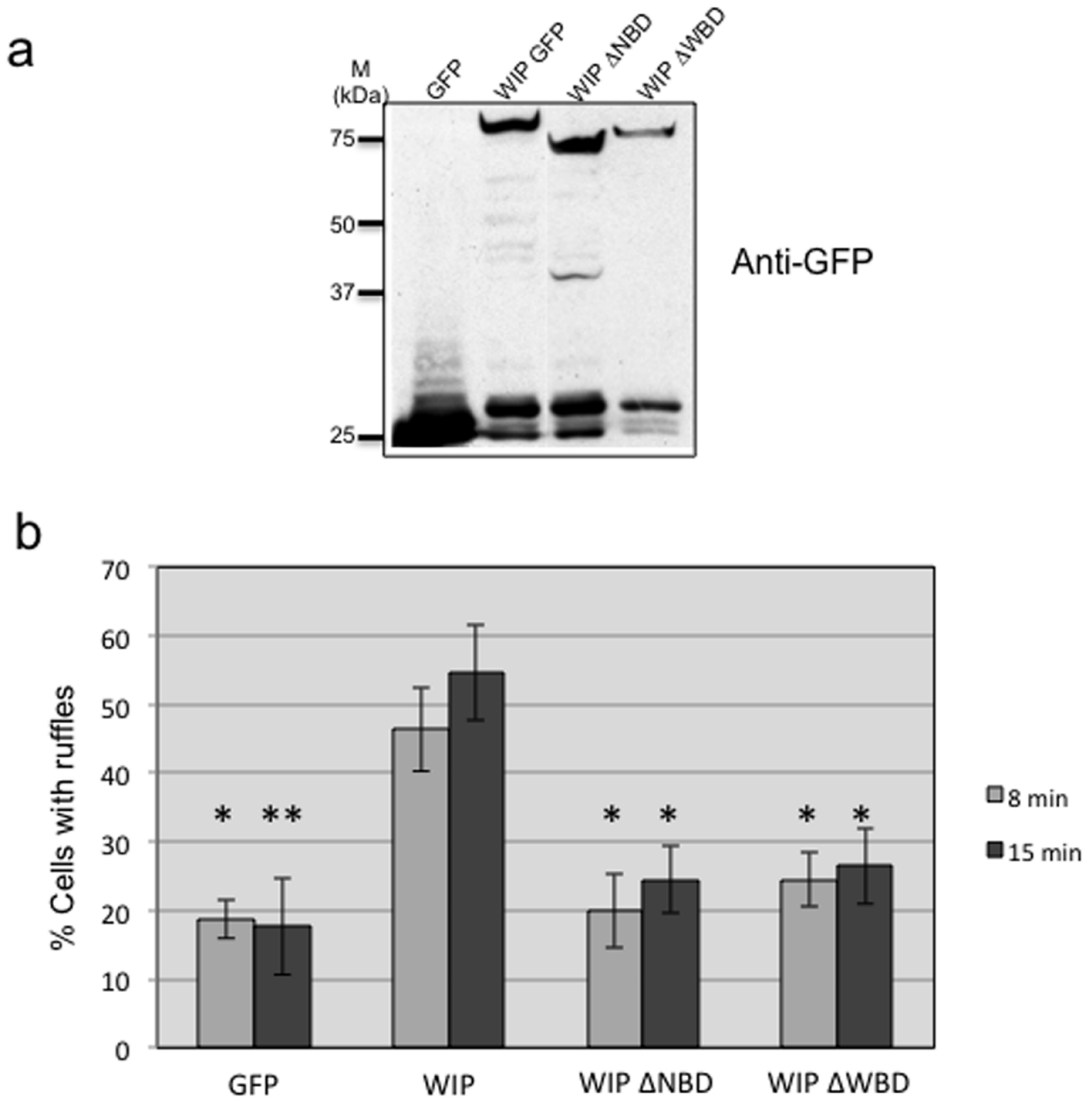
Nck and N-WASP binding to WIP contribute to dorsal ruffle formation induced by PDGF-AA stimulation. **a** WIP^−/−^ primary murine fibroblasts were transduced with recombinant lentivirus coding for GFP, WIP-GFP, WIPΔNBD-GFP (missing the Nck binding domain) or WIPΔWBD-GFP (missing the N-WASP binding site). Soluble protein extracts were subjected to western blot analysis, using anti-GFP as probe, and developed with ECL detection kit. **b** Transduced WIP^−/−^ murine fibroblasts were serum starved and stimulated with PDGF-AA for increasing times (8 and 15 min). Fixed and permeabilised cells were stained with anti-cortactin and FITC-secondary antibody and imaged in a Zeiss microscope. The percentage of GFP-positive cells forming dorsal ruffles after PDGF-AA stimulation is plotted against incubation times. One way ANOVA test and Test of Tukey * p<0,05; ** p<0,001.

WIP binds to proteins involved in dorsal ruffle formation such as actin, cortactin, mAbp1, Nck and N-WASP [14], [39], [36]. The interaction between WIP and actin [14] or mAbp1 is important to dorsal ruffle formation but the WIP-cortactin interaction is dispensable [39]. There remains a gap in our understanding of the contribution of the WIP-Nck-N-WASP complex to ruffle generation. To gain further insight into how WIP interactions regulate dorsal ruffle formation, WIP^−/−^ fibroblasts were transduced with recombinant lentivirus expressing GFP, full length WIP-GFP or WIP mutants deleted of the Nck-binding site domain (a.a. 321–415; WIPΔNBD) or N-WASP-binding domain (a.a. 450–503; WIPΔWBD) [40] also fused to GFP. Equivalent expressed protein was confirmed by Western blot analysis (Figure 7a) and infected cells were treated with PDGF-AA to induce dorsal ruffle formation (Figure 7b). Deficient dorsal ruffle formation in WIP-null cells was seen to be restored by expression of wild-type WIP-GFP but not by the WIP mutants WIPΔNBD or WIPΔWBD, suggesting that WIP binding to Nck and N-WASP is essential for this process (Figure 7b). These results show that WIP complexes with Nck and N-WASP to contribute to dorsal ruffle formation after PDGF-AA stimulation.

### WIP binding to Nck but not to N-WASP is required for chemotaxis towards PDGF-AA

**Figure 8:**
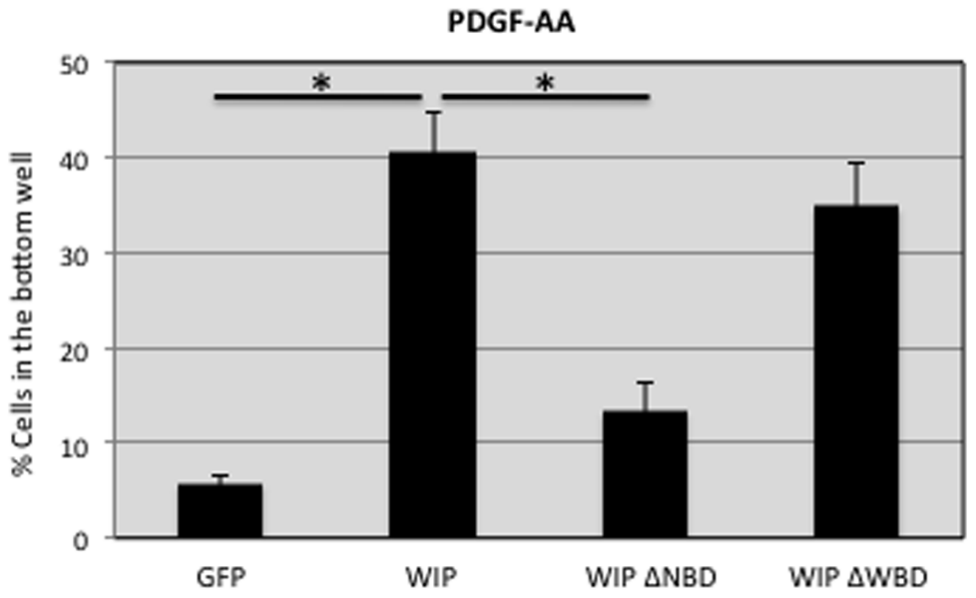
Nck binding to WIP contributes to chemotaxis towards PDGF-AA. WIP^−/−^ murine fibroblasts were transduced with recombinant lentivirus engineered to express control GFP or fusion constructs including WIP, WIPΔNBD or WIPΔWBD. Fibroblasts were loaded onto the upper chamber of the Transwell and the percentage of GFP-expressing cells chemotacting towards 50 ng/ml PDGF-AA was calculated. * p<0,05.

Dorsal ruffle formation has been linked to fibroblast migration [41]. In this report we demonstrate that WIP deficiency perturbs both fibroblast chemotaxis and dorsal ruffle generation. To discover whether WIP domains involved in chemotaxis overlapped with those whose contribution to ruffle formation was identified, we quantified the percentage of cells translocating to the PDGF-AA-enriched lower chamber of a Transwell after WIP^−/−^ fibroblasts were transduced with recombinant lentivirus expressing GFP, full length WIP-GFP, WIPΔNBD or WIPΔWBD (Figure 8). Chemotactic response to PDGF-AA by WIP-deficient cells was rescued after expression of wild-type WIP-GFP and WIP-ΔWBD but not by WIP mutants lacking the capability to bind Nck (WIP-ΔNBD). These data strongly point to Nck binding to WIP being essential for the chemotactic response (Figure 8).

## Discussion

In this study we identify WIP as an essential component for cell persistence during amoeboid migration of splenic B lymphocytes and mesenchymal migration of lung fibroblasts. We also show that WIP localizes to and regulates lamellipodium formation in CXCL13-treated B cells and circular dorsal ruffle formation in PDFG-AA-stimulated fibroblasts. Moreover, we conclude that WIP-dependent signals required for dorsal ruffle formation and efficient fibroblast chemotaxis towards PDGF-AA involve WIP binding to Nck, an adaptor protein and N-WASP activator. Finally, we demonstrate that dorsal ruffle formation is not a pre-requisite for proper chemotactic responses since WIP binding to N-WASP contributes to dorsal ruffle formation but is not essential for chemotaxis of primary fibroblasts towards PDGF-AA.

B cells lacking WIP have defects in the actin cytoarchitecture [30], though they do not show distinct morphology from control cells in the steady state (Figure S1). However, the absence of WIP diminishes chemokine-triggered cell polarization, migration, and F-actin-rich lamellipodium formation in B cells. These alterations may be related to low WASP levels due to its degradation in the absence of WIP with a knock-on effect on the activation of the Arp2/3 complex [42]. In addition, other WASP-independent WIP functions on the actin cytoskeleton may be implicated [43]. The decreased B cell chemotactic response to a gradient of CXCL13 chemokine together with the lower values of the persistence index obtained in the planar membranes, where random migration takes place, point to an important role for WIP in regulating directional persistence in amoeboid motility. In support of our findings, it has been shown that WASP-deficient macrophages have reduced directional migration [20]. The defects in cell behaviour derived from WIP deficiency may compromise B cell localization and pathogen searching, thus, B cell function. In fact, defective B cell migration may underline the severe reduction of the area containing follicular B cells (B220^+^) observed in the spleen of WIP−/− mice [29] and contribute to the immune disorder that WIP null mice develop [29]. It would be interesting to study the migratory functionality of B cells from the recently identified immunedeficient patient carrying a WIP mutation [44].

Our previous results have shown that actin distribution in serum-starved primary lung fibroblasts from WIP^−/−^ mice was similar to control fibroblasts from WIP^+/+^ mice [14]. In this report we have compared the morphology of growing fibroblasts and our study indicates that in the presence of serum WIP does not contribute to gross differences in overall cellular size, rugosity or shape (Figure 3b–c), confirming that no significant morphological differences are detected in growing fibroblasts (up to 12 passages) in the absence of WIP. These results demonstrate that WIP is not essential for cell architecture in primary murine fibroblasts. Our data also support previous reports [28], [45] indicating that WIP does not regulate N-WASP stability since, in contrast to WASP in hematopoietic cells, N-WASP levels are not modified in WIP^−/−^ fibroblasts (Figure 3a). A recent report described that siRNA-mediated reduction of WIP levels in mouse embryonic fibroblasts (MEFs) decreases N-WASP levels [36]. This disagreement may be due to differences in cell type (murine fibroblasts versus MEFs) or to an adaptative response favored by permanent and total loss of WIP expression in WIP-deficient lung fibroblasts compared to the partial loss of WIP in interfered MEFs.

Fibroblasts migrate on 2D surfaces by forming actin-rich lamellipodia at the leading edge of the cell [46]. Lamellipodia and ruffles are widely believed to be critical for directional cell motility and their generation depends on Arp2/3 activity [47]. However, conflicting data on the contribution of Arp2/3 to directional migration appears in the literature: Whereas some publications support the argument that the Arp2/3 complex is required for lamellipodia extension and directional fibroblast cell migration [48], other reports indicate that Arp2/3-depleted fibroblast respond normally to shallow gradients of PDGF, indicating that lamellipodia are not required for chemotaxis [49]. Our results support the latter idea: actin-rich membrane protrusions such as circular dorsal ruffles are dispensable for PDGF-AA-induced chemotaxis since fibroblasts reconstituted with a WIP mutant that does not bind N-WASP (WIP-ΔWBD) have significantly decreased formation of dorsal ruffles but intact chemotactic capability (Figures 7 and 8). Many reports describe PDGF-BB stimulation as a first step for dorsal ruffle formation [50]. However, to our knowledge, this is the first work defining a specific role for PDGF-AA (and by extension PDGFRα stimulation) in the formation of actin-rich dorsal ruffles. Endocytosis of the PDGFR promotes actin remodelling and cell migration. PDGFR uptake has been shown to be delayed in WIP KD cells 60–120 min after PDGF-BB stimulation and not affected at earlier times (15 min) [36]. Similarly, PDGFRα endocytosis in WIP^−/−^ fibroblasts is not modified 15 min after stimulation (Figure 6f), time points that match with the dorsal ruffle response. Therefore, the abnormal dorsal ruffling is unlikely to be due to reduced PDGFRα uptake following ligand binding.

In this report, we provide the first evidence for WIP/Nck/N-WASP participation in PDGF-AA-mediated ruffle generation and of WIP/Nck, but not N-WASP, in PDGF-AA-mediated chemotaxis in 2D. The latter results are in accordance with previous reports showing that N-WASP contributes to dorsal ruffle formation but not to 2D migration [51]. N-WASP(^−/−^) fibroblast-like cells generate aberrant dorsal ruffles; highly unstable, severely depleted of Arp2/3 complex, and diminished in size [15]. In addition, expression of a N-WASP truncation mutant that cannot bind Arp2/3 complex block the formation of these structures [15]. These results suggest that N-WASP and Arp2/3 complex are part of a multiprotein assembly important for the generation of PDGFAA-induced dorsal ruffles. A similar correlation between Nck and the formation of dorsal ruffles has been observed [52]. These observations clearly put Nck and its binding partners in a common pathway in the formation of dorsal ruffles. Our data support this idea and add N-WASP interaction with WIP as an essential step in the process. Moreover, WIP and Nck appear as essential components of the complex that regulates dorsal ruffle formation. These results place WIP within a biochemical pathway (PDGFR/Nck-WIP-N-WASP/Arp2/3-actin) that links growth factor stimulation to dynamic actin changes that are involved in cell motility and morphological plasticity. Moreover, these and previous results support the hypothesis that WIP might function as a scaffolding molecule, with the potential to influence both actin polymerization and the assembly of actin filaments into higher-order arrays involved in adhesion and migration. Our data fits well with a recently proposed model arising from both computational simulations and experimentation, in which the density of Nck molecules in multicomponent aggregates is a critical determinant of actin polymerization with a Nck/N-WASp/Arp2/3 stoichiometry of 4:2:1 [53]. The two N-WASP molecules would provide two Nck molecules and the other two Nck proteins required to reach a total of four, would come from binding to WIP. Finally, we have confirmed WIP-cherry location in dorsal ruffles in reconstituted WIP^−/−^ murine lung fibroblasts (Figure 6c) in agreement with previous studies showing endogenous WIP in dorsal ruffles and overexpressed in 3T3 fibroblasts [14], [39]. Moreover, we describe the preferential distribution of WIP at the lamellipodial protrusions formed by B lymphocytes during their amoeboid migration.

## Supporting Information

Figure S1 **Phenotypic analysis of WIP^−/−^ B cells.** **a** Freshly isolated B cells from WIP^+/+^ and WIP^−/−^ mice were analysed for cell size (Forward scatter), cell complexity (Side scatter), and expression of the cell surface markers CD19 and IgM by flow cytometry. Profiles of a representative experiment are shown; the purity of the purified B cell fraction was 95% for WIP^+/+^ and 85% for WIP^−/−^. **b** Lysates of WIP^+/+^ and WIP^−/−^ B cells were assessed for WIP, WASP, Nck and tubulin protein expression levels by western-blot; numerical values below bands correspond to expression levels for each protein in WIP^−/−^ B cells in comparison to WIP^+/+^ B cells.
(TIF)

Figure S2 **Predominant localization of WIP at the lamella of migratory B cells.** 2PK3 B cell line was transiently transfected with full-length WIP-GFP expression vector; 24 h after, cells were settled on planar membranes and monitored for migration by real-time microscopy. DIC, WIP-GFP and IRM time-frame images at the contact plane of a representative B cell with the 2D substrate are shown; white arrow, accumulation of WIP-GFP at the lamella of the cell.
(PDF)

Figure S3 **WIP deficiency reduces persistence but not velocity during chemotaxis towards serum.** Control (WIP^+/+^) and WIP^−/−^ murine fibroblasts were assayed for chemotaxis towards 15% serum in Dunn chambers. **a** Individual cell track with black dots at the end point of cell displacement. **b** Individual cell velocity profile (upper) and mean velocity values (lower) calculated by Mathematica software. **c** Persistence profiles of individual cells (each line represents a single cell) calculated by Mathematica software. Arbitrary units, a.u.
(PPT)

Figure S4 **PDGF-AA-induced dorsal ruffle formation is diminished in WIP** ^−**/**−^ **fibroblasts.** **a** Control (WIP^+/+^) and WIP^−/−^ primary murine fibroblasts were serum starved over night (0 min) or serum starved and stimulated with PDGF-AA for increasing times (8 and 15 min). Fixed and permeabilised cells were stained with TRITC-phalloidin to label actin filaments and imaged in a Zeiss microscope. Dorsal ruffles are indicated by white arrows. **b** WIP^−/−^ primary fibroblasts were lentivirally transduced to express control cherry or WIP-cherry, starved and incubated with PDGF-AA for 8 or 15 min. Fixed and permeabilised cells were stained with FITC-phalloidin to label actin filaments and imaged in a Zeiss microscope.
(PPTX)

Video S1 **Migration of wild type and WIP-deficient B cells.** Purified WIP^+/+^ (CFSE-labeled; green) and WIP^−/−^ (SNARF-1 labeled; red) B cells, mixed in a 1∶1 ratio, migrating on ICAM-1-containing planar membranes coated with CXCL13. DIC (left panel), fluorescence (CFSE, SNARF-1; middle panel) and IRM (right panel) images over time (6 frames/second) are shown. The tracks followed by migratory B cells (IRM positive) are highlighted with the dragon tails (green/red lines).
(MOV)

Video S2 **WIP localization in motile B cells.** Migration of a representative 2PK3 B cell transfected with WIP-GFP construct on ICAM-1-containing planar membranes coated with CXCL13. DIC (left panel), WIP-GFP fluorescence (middle panel) and IRM (right panel) images at the contact plane of the 2PK3 B cell with the target membrane over time (2 frames/second) are shown.
(MPG)

Video S3 **Directional migration towards serum of murine fibroblasts in Dunn chambers.** Murine lung fibroblasts were seeded onto 18-mm square glass coverslips and grown for 12–24 h. Cells were serum starved for 8 h and exposed to a serum gradient (15% FCS in the outer well). Cells were filmed at 37°C on Olympus IX50 Inverted microscopes fitted with phase-contrast optics, heated stages, and heated chambers. Frames were filmed using a CCD camera (Hitachi) every 5 min for 8 h using Acquisition Manager software from Kinetic Imaging (Wirral, UK).
(MPG)

Video S4 **Directional migration towards serum of WIP** ^−**/**−^ **murine fibroblasts in Dunn chambers.** WIP^−/−^ murine lung fibroblasts were seeded onto 18-mm square glass coverslips and grown for 12–24 h. Cells were serum starved for 8 h and exposed to a serum gradient (15% FCS in the outer well). Cells were filmed at 37°C on Olympus IX50 Inverted microscopes fitted with phase-contrast optics, heated stages, and heated chambers. Frames were filmed using a CCD camera (Hitachi) every 5 min for 8 h using Acquisition Manager software from Kinetic Imaging (Wirral, UK).
(MPG)
