# Supplementary figures and images for: WIP Regulates Persistence of Cell Migration and Ruffle Formation in Both Mesenchymal and Amoeboid Modes of Motility
Source: PLoS One. 2013 Aug 7;8(8):e70364. doi: 10.1371/journal.pone.0070364 (PMC3737202; doi:10.1371/journal.pone.0070364)

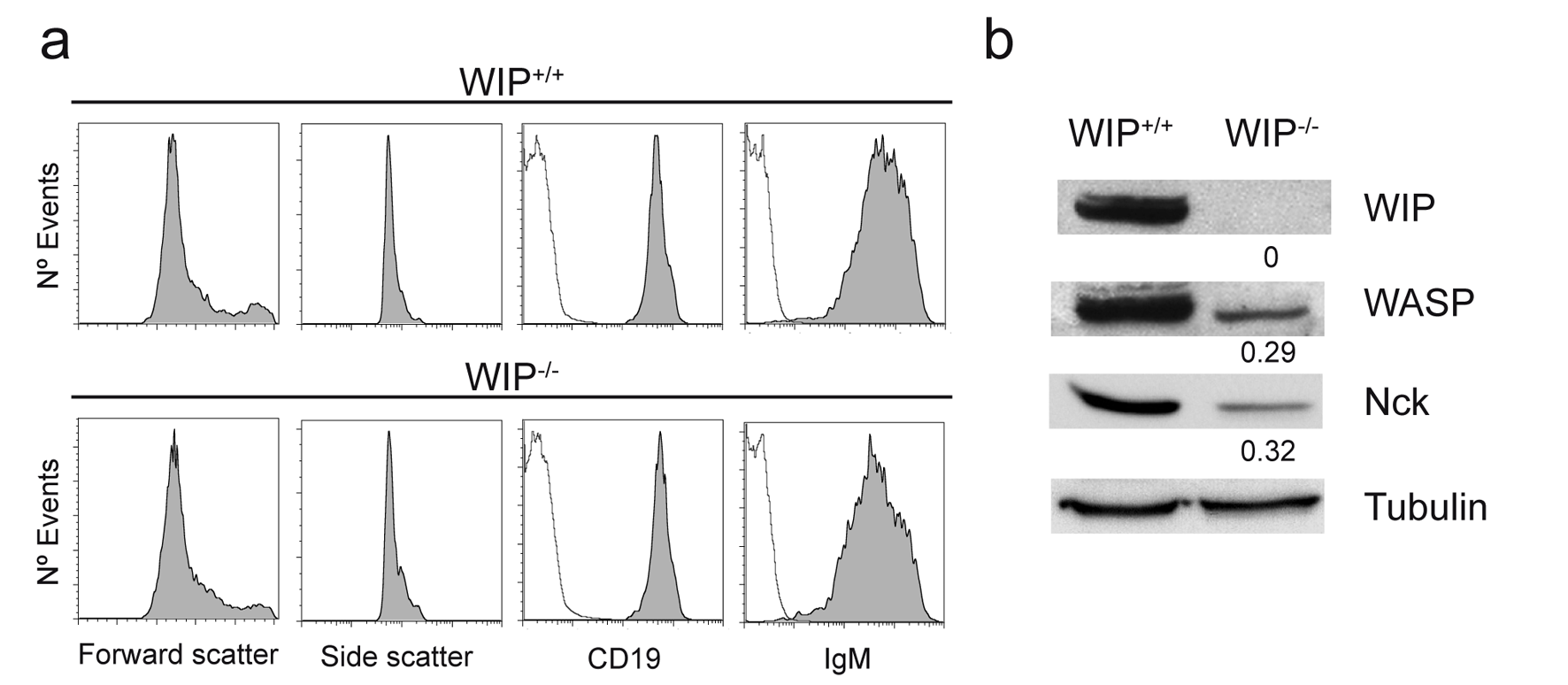

Supplement: Figure S1 — Phenotypic analysis of WIP−/− B cells. a Freshly isolated B cells from WIP+/+ and WIP−/− mice were analysed for cell size (Forward scatter), cell complexity (Side scatter), and expression of the cell surface markers CD19 and IgM by flow cytometry. Profiles of a representative experiment are shown; the purity of the purified B cell fraction was 95% for WIP+/+ and 85% for WIP−/−. b Lysates of WIP+/+ and WIP−/− B cells were assessed for WIP, WASP, Nck and tubulin protein expression levels by western-blot; numerical values below bands correspond to expression levels for each protein in WIP−/− B cells in comparison to WIP+/+ B cells. (TIF) [file pone.0070364.s001.tif]

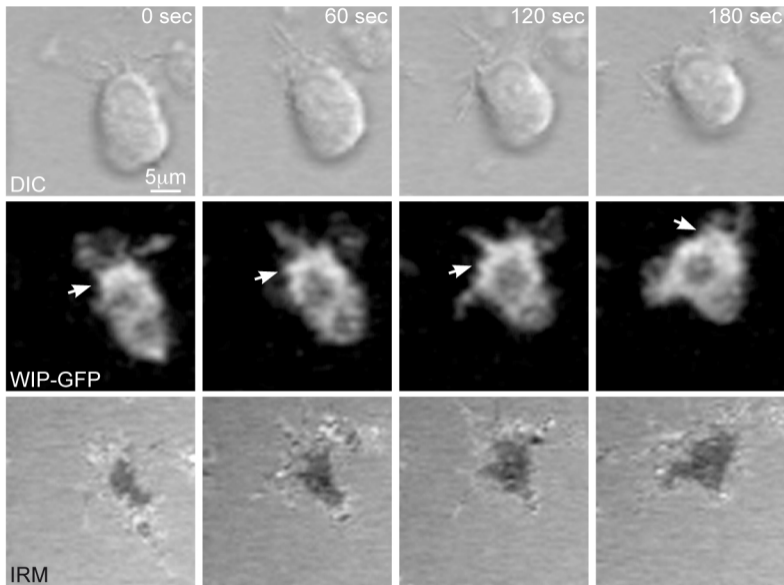

Supplement: Figure S2 — Predominant localization of WIP at the lamella of migratory B cells. 2PK3 B cell line was transiently transfected with full-length WIP-GFP expression vector; 24 h after, cells were settled on planar membranes and monitored for migration by real-time microscopy. DIC, WIP-GFP and IRM time-frame images at the contact plane of a representative B cell with the 2D substrate are shown; white arrow, accumulation of WIP-GFP at the lamella of the cell. (PDF) [file pone.0070364.s002.pdf]

## Slide 1
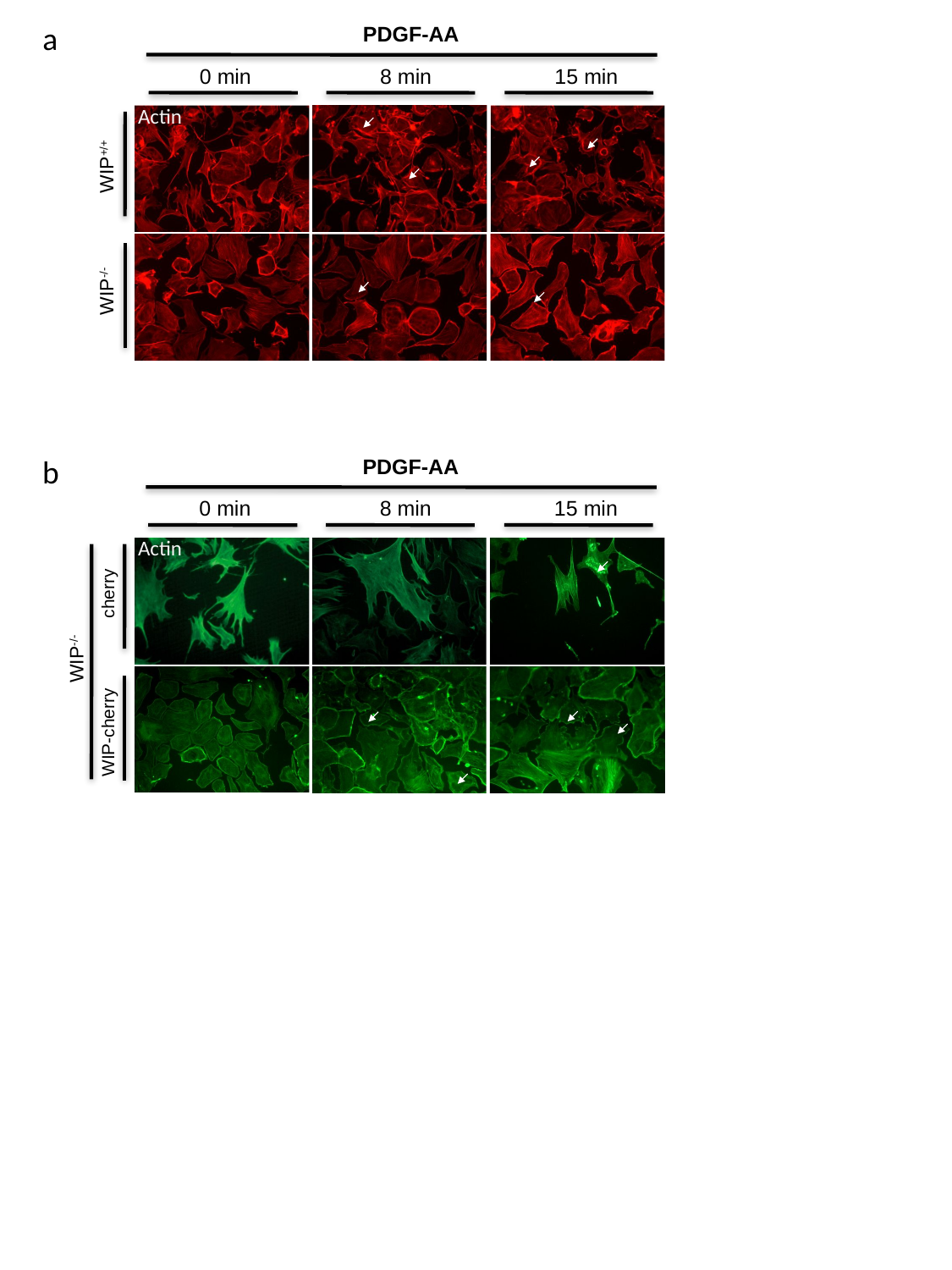

a
PDGF-AA
8 min
 15 min
0 min
Actin
 WIP+/+
 WIP-/-
b
PDGF-AA
8 min
 15 min
0 min
Actin
 cherry
 WIP-/-
WIP-cherry

Supplement: Figure S4 — PDGF-AA-induced dorsal ruffle formation is diminished in WIP −/− fibroblasts. a Control (WIP+/+) and WIP−/− primary murine fibroblasts were serum starved over night (0 min) or serum starved and stimulated with PDGF-AA for increasing times (8 and 15 min). Fixed and permeabilised cells were stained with TRITC-phalloidin to label actin filaments and imaged in a Zeiss microscope. Dorsal ruffles are indicated by white arrows. b WIP−/− primary fibroblasts were lentivirally transduced to express control cherry or WIP-cherry, starved and incubated with PDGF-AA for 8 or 15 min. Fixed and permeabilised cells were stained with FITC-phalloidin to label actin filaments and imaged in a Zeiss microscope. (PPTX) [file pone.0070364.s004.pptx]
