# Supplementary material for: WIP Regulates Persistence of Cell Migration and Ruffle Formation in Both Mesenchymal and Amoeboid Modes of Motility
Source: PLoS One. 2013 Aug 7;8(8):e70364. doi: 10.1371/journal.pone.0070364 (PMC3737202; doi:10.1371/journal.pone.0070364)
Supplement: Figure S3 — WIP deficiency reduces persistence but not velocity during chemotaxis towards serum. Control (WIP+/+) and WIP−/− murine fibroblasts were assayed for chemotaxis towards 15% serum in Dunn chambers. a Individual cell track with black dots at the end point of cell displacement. b Individual cell velocity profile (upper) and mean velocity values (lower) calculated by Mathematica software. c Persistence profiles of individual cells (each line represents a single cell) calculated by Mathematica software. Arbitrary units, a.u. (PPT) [file pone.0070364.s003.ppt]

## Slide 1
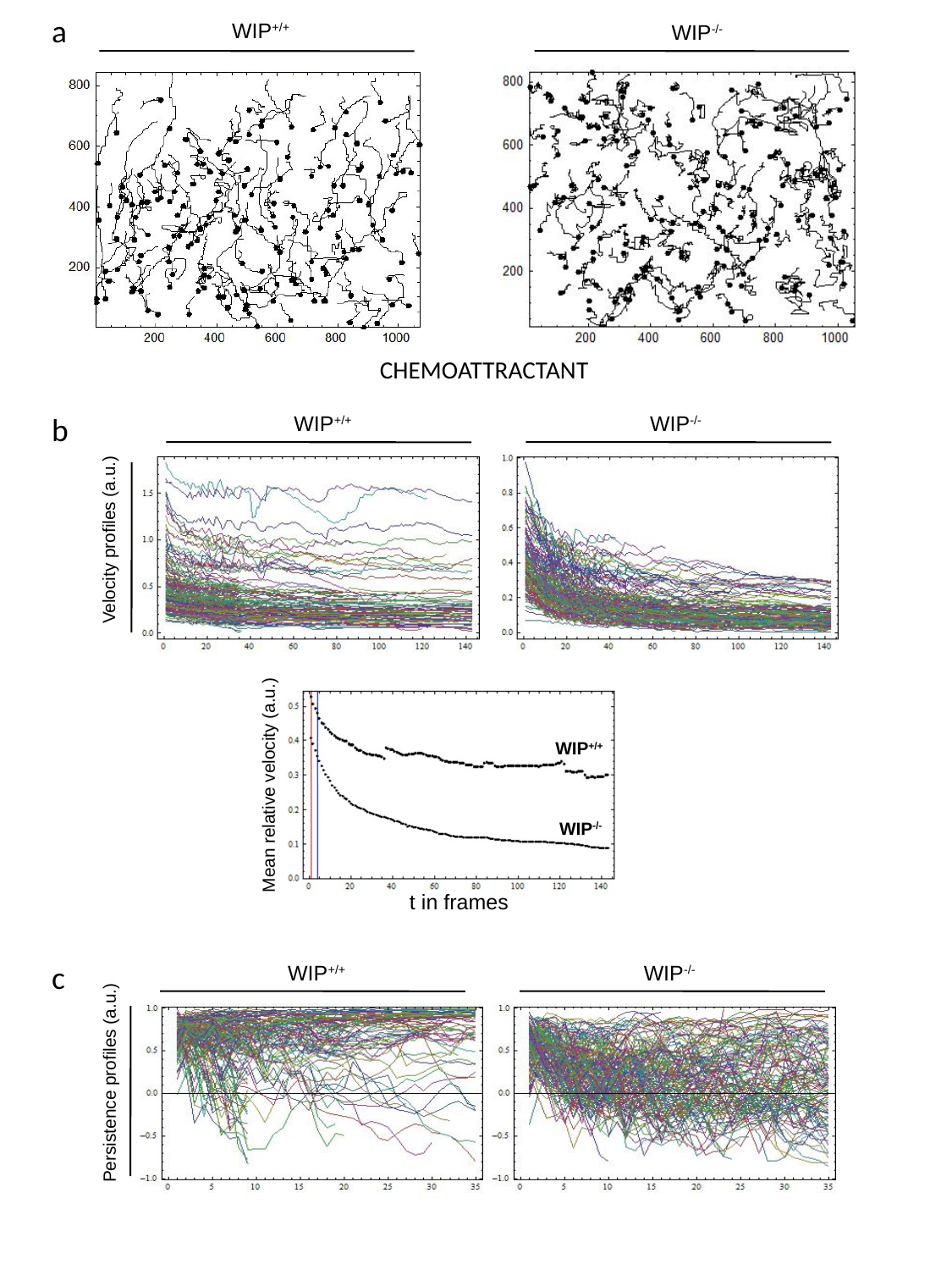

a
WIP+/+
WIP-/-
CHEMOATTRACTANT
b
WIP+/+
WIP-/-
Velocity profiles (a.u.)
WIP+/+
Mean relative velocity (a.u.)
WIP-/-
t in frames
WIP+/+
WIP-/-
Persistence profiles (a.u.)
c
